# Chyle leakage after laparoscopic cholecystectomy in a patient with duplicated cystic ducts: A case report and literature review

**DOI:** 10.1097/MD.0000000000039982

**Published:** 2024-10-04

**Authors:** Danfeng Shen, Yingchao Lu, Peng Chang, Hongxing Xu

**Affiliations:** a Department of General Surgery, Taicang Affiliated Hospital of Soochow University, Suzhou, P.R. China.

**Keywords:** case report, cholelithiasis, chyle leakage, duplicated cystic duct, laparoscopic cholecystectomy

## Abstract

**Rationale:**

Laparoscopic cholecystectomy (LC) is widely performed as a standard treatment for cholelithiasis, and chyle leakage after LC has rarely been reported. Duplicated cystic ducts draining a single gallbladder is an extraordinarily rare variation.

**Patient concerns:**

We presented a case of chyle leakage after LC in a 53-year-old female with a rare variation of duplicated cystic ducts.

**Diagnoses:**

Chyle leakage and duplicated cystic ducts.

**Interventions:**

Conservative treatment including lipid intake and constant drainage.

**Outcomes:**

After 24 days of conservative treatment, the patient recovered and had no further troubles during the 3-month follow-up.

**Lessons:**

There may be a potential relationship between anatomic variants of the extrahepatic bile ducts and those of the lymphatic system. When anatomic variations of the extrahepatic bile ducts are encountered, vigilance for lymphatic system injuries is as important as vigilance for bile duct injuries. Conservative therapy is the first choice for postoperative chyle leakage, and surgical intervention should be considered in cases with high-volume chyle leakage.

## 1. Introduction

Although laparoscopic cholecystectomy (LC) is considered as the gold standard for the treatment of cholelithiasis,^[1]^ it still faces challenges such as anatomic variations of extrahepatic bile duct.^[2]^ Duplicated cystic ducts draining a single gallbladder is a rare variation,^[3]^ with <30 cases have been reported worldwide. As a well-established surgical procedure, LC has a very low mortality and complication rate,^[4]^ and reports of chyle leakage after LC have rarely been reported. We present a rare case of duplicated cystic ducts draining a single gallbladder that developed chyle leakage after LC. The case is unique in that it is associated with 2 rare conditions, which presents a double challenge. The relationship between chyle leakage and variations of extrahepatic bile duct is a clinical dilemma.

## 2. Case report

**Figure 1.**
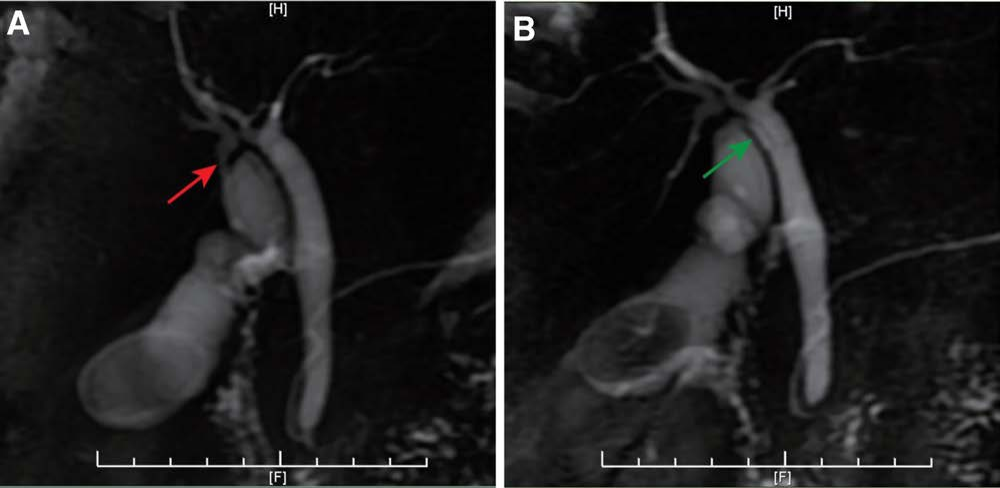
Duplicated cystic ducts were suspected based on the preoperative MRCP images. (A) The suspected accessory cystic duct (red arrow). (B) The cystic duct (green arrow). MRCP = magnetic resonance cholangiopancreatography.

A 53-year-old female underwent elective LC for symptomatic cholelithiasis. The patient had no significant medical and surgery history. Ultrasound revealed multiple gallstones and a normal common bile duct. Physical examination and laboratory tests including liver function tests were unremarkable. Preoperative magnetic resonance cholangiopancreatography (MRCP) showed an abnormal biliary system with a suspected accessory cystic duct (Fig. 1).

**Figure 2.**
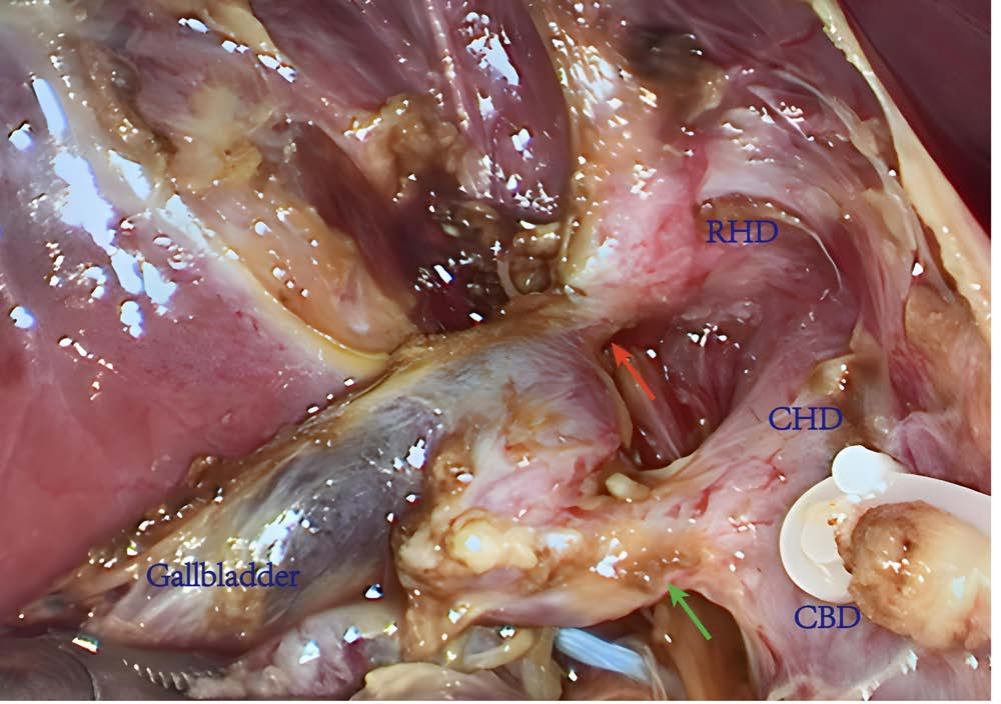
The intraoperative findings revealed a duplicated cystic duct (the gallbladder artery had been clipped and divided): the accessory cystic duct (red arrow); the cystic duct (green arrow). CBD = common bile duct, CHD = common hepatic duct, RHD = right hepatic duct.

**Figure 3.**
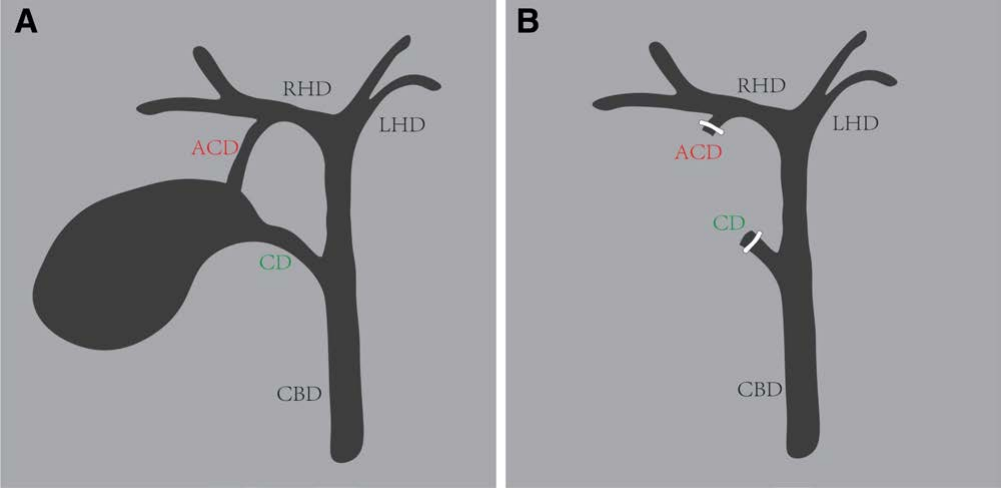
The schematic diagram of biliary system and surgical procedure in the present case. ACD = accessory cystic duct, CBD = common bile duct, CD = cystic duct, LHD = left hepatic duct, RHD = right hepatic duct.

LC was performed using conventional 3-hole approach, and the extrahepatic bile duct was carefully dissected intraoperatively. A smaller caliber (2–3 mm) accessory cystic duct was identified, extending from the neck of the gallbladder to the right hepatic duct (RHD) (Fig. 2). As the ability of performing intraoperative cholangiography (IOC) was not available in our center, an experienced consultant hepatobiliary surgeon was invited to provide further assistance. Based on the preoperative MRCP images and the intraoperative findings, a rare anatomic variation of duplicated cystic ducts was finally confirmed and duplicated cystic ducts were cut off separately. The schematic diagram of biliary system and surgical procedure are shown in Figure 3. After removal of the gallbladder from the liver bed, a 16-Fr negative pressure drain was placed in the gallbladder fossa. Postoperative supportive therapy was given and oral intake was gradually resumed later.

**Figure 4.**
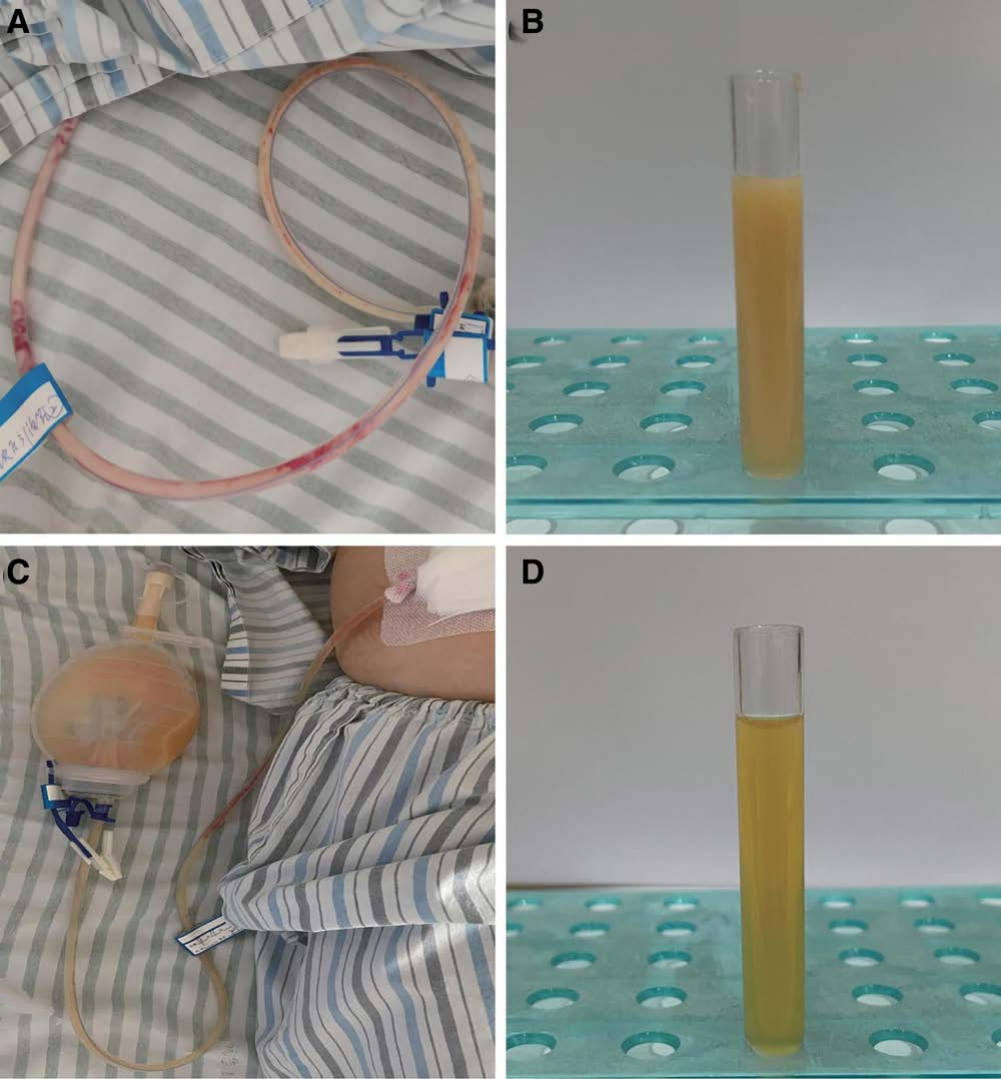
Chylous ascites in the drain. (A, B) The ascites on the second day. (C, D) The ascites after conservative therapy on the seventh day.

**Figure 5.**
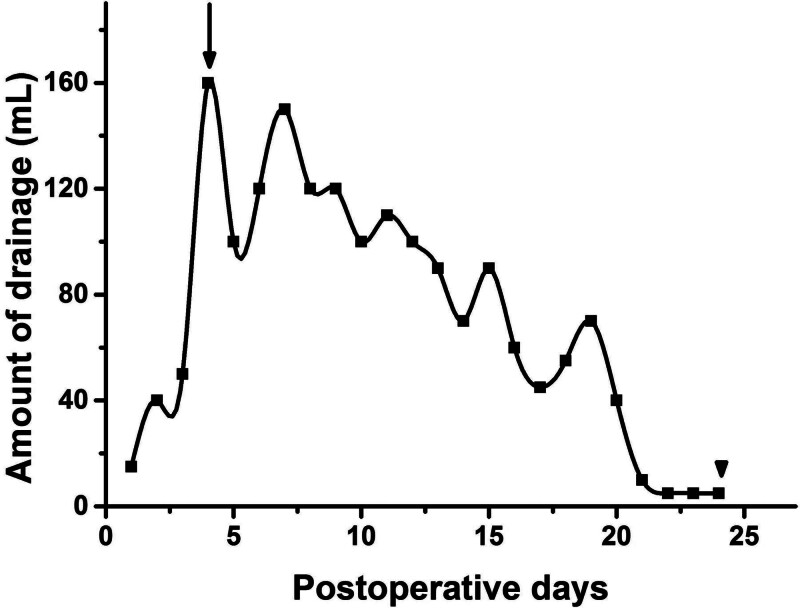
Serial changes in the amount of drainage: maximum amount of drainage (arrow); removal the drainage tube (arrowhead).

Later on the day of surgery, a small amount of milky white odorless ascites was found in the drain, which turned into a yellow turbid fluid the next day (Fig. 4). The initial drainage of the ascites was approximately 20 to 40 mL/d, but increased day by day and maintained at 100 to 120 mL/d after the seventh postoperative day. The laboratory analysis showed the level of triglycerides in the ascites (20.40 mmol/L) was more than 10 times that of serum triglycerides (1.43 mmol/L), which was consistent with chyloperitoneum. The patient did not show any significant discomfort and was treated conservatively. After controlling lipid intake, the ascites gradually became less turbid (Fig. 4) and the patient was discharged on the tenth day postoperative. After discharge, the patient was followed up continuously, and the amount of drainage was recorded daily (Fig. 5). The drain was removed when the amount of drainage was <5 mL for 3 consecutive days (on the 24th day postoperative). The patient had no further troubles during the 3-month follow-up.

## 3. Discussion

Duplicated cystic ducts draining a single gallbladder are extraordinarily rare and can be categorized into 3 types: type “Y,” type “H” and trabecular type.^[5]^ The present case belongs to type “H” with an accessory cystic duct drainage into the RHD. The schematic diagram of biliary system in this case is shown in Figure 3. The first challenge we faced in this case was the absence of IOC to confirm the diagnosis and the duplicated type further. However, endoscopic retrograde cholangiopancreatography, MRCP or IOC are not foolproof techniques against bile duct injurys.^[6,7]^ Constant vigilance for rare anatomic variants, careful dissection and the assistance of experienced consultant hepatobiliary surgeons are equally crucial to avoid bile duct injurys.^[6]^

Chyle leakage after cholecystectomy is an extremely rare complication. To the best of our knowledge, only five such cases were reported previously^[8–12]^ (Table 1). Chyle leakage should be suspected when white or yellow milky fluid is present in the drain, and it can be diagnosed by fluid triglyceride levels >110 mg/dL (equivalent to 1.24 mmol/L) or 2 to 8 times the serum triglyceride level.^[8]^ In the current case, we did not perform lymphangiography (the gold standard in the diagnosis of lymphatic leakage) or other additional iconography tests because the diagnosis was clearly confirmed by the nature and the triglycerides level of the fluid.

**Table 1:** Literature review of the reported cases of chyle leakage after cholecystectomy.

Case	Age (yr)	Gender	Surgical procedure	Drainage fluid* (mL/d)	Management	TPN	Total recovery time (d)	Year	Reference
1	31	Female	LC	1500	Laparoscopic reexploration	Yes	Approximately 30	2006	Jensen and Weiss^[8]^
2	69	Male	LC	100	Conservative treatment only	No	17	2009	Huang et al^[9]^
3	26	Female	LC	340	Conservative treatment only	No	7	2010	Gogalniceanu et al^[10]^
4	37	Male	OC	950	Conservative treatment only	No	17	2011	Cheung et al^[11]^
5	40	Male	LC	9000	Laparoscopic reexploration	Yes	210	2017	Yao et al^[12]^
6	46	Female	LC	160	Conservative treatment only	No	24	2023	The present case

LC = laparoscopic cholecystectomy, OC = open cholecystectomy, TPN = total parenteral nutrition.

* Maximum amount of drainage.

Lymphatic networks in the liver is consisting of lymphatic vessels along the portal tract (deep lymphatic system) and within the collagenous Glisson capsule (superficial lymphatic system).^[13]^ Cheung et al^[11]^ thought that a derangement of anatomy must exist, making it more vulnerable to the development of chylous ascites. Since an anatomic variation of extrahepatic bile duct was present in our case, we clearly dissected the hilar bile duct and revealed the initiation of the RHD, which may have unknowingly caused an abnormal superficial lymphatic system injury. Therefore, we postulate that there may be a potential relationship between the anatomic variation of extrahepatic bile duct and the anatomic variation of the lymphatic system. The limitation of our case was the absence of lymphangiography to confirm our speculation, which was the second challenge we faced in this case.

Once a diagnosis of chyle leakage is made, it should be treated aggressively. Prophylactic antibiotics are the consensus for preventing secondary infections in the abdominal cavity.^[14]^ Keeping the drain unobstructed and a low-fat and high-protein diet or total parenteral nutrition (TPN) are well recommended.^[15]^ The difficulty in treating chyle leakage is that the optimal reoperation time and reexploration method are hard to determined and no consensus has been established. Low-volume chyle leakage (<1000 mL/d) usually resolves spontaneously with conservative therapy and does not necessarily require the use of TPN,^[9–11]^ while high-volume chyle leakage (>1000 mL/d) may not be successfully cured without TPN and further surgical treatment.^[8,12]^ In another published literature, this low-volume or high-volume standard was suggested as 500 mL/d.^[14]^ The choice of surgical reexploration method should be individualized according to the patient’s physical condition, the initial mode of surgery, the underlying disease, and the severity of chyle leakage.^[9]^ According to previous literatures,^[8,12]^ laparoscopic reexploration seems to be more recommended, and by which it is easy to obtain a good view and directly locate the exact position of the chyle leakage.

## 4. Conclusion

When anatomic variations of the extrahepatic bile ducts are detected, we must remain vigilant because not only the bile ducts but also the lymphatic vessels may be damaged. Conservative therapy, including prophylactic antibiotics, unobstructed drainage, and a low lipid diet, is the first choice for postoperative chyle leakage. TPN may not be necessary for low-volume chyle leakage, whereas high-volume chyle leakage may require TPN and further surgical intervention.

## Author contributions

**Funding acquisition:** Danfeng Shen.

**Methodology:** Danfeng Shen.

**Project administration:** Danfeng Shen.

**Writing—original draft:** Danfeng Shen, Yingchao Lu.

**Visualization:** Yingchao Lu, Peng Chang.

**Data curation:** Peng Chang.

**Supervision:** Hongxing Xu.

**Writing—review & editing:** Hongxing Xu.
